# Effects of caffeine supplementation on anaerobic power and muscle activity in youth athletes

**DOI:** 10.1186/s13102-023-00805-1

**Published:** 2024-01-19

**Authors:** Leila Ghazaleh, Anita Enayati, Maryam Delfan, Sobhan Bamdad, Ismail Laher, Urs Granacher, Hassane Zouhal

**Affiliations:** 1 Department of Exercise Physiology, Faculty of Sport Sciences, Alzahra University, Tehran, Iran; 2 Department of Biomedical Engineering, Faculty of Engineering, Shahed University, Tehran, Iran; 3 Department of Anesthesiology, Pharmacology, and Therapeutics, Faculty of Medicine, University of British Columbia, Vancouver, Canada; 4 Department of Sport and Sport Science, Exercise and Human Movement Science, University of Freiburg, Freiburg, Germany; 5 Univ Rennes, M2S (Laboratoire Mouvement), EA 1274, Sport, Rennes, Santé F-35000 France; 6 Institut International des Sciences du Sport (2I2S), Irodouer, 35850 France

**Keywords:** Supplements, Acute effect, Central fatigue, Wavelet, Power frequency

## Abstract

This study aimed to investigate the effects of caffeine ingestion on anaerobic performance and muscle activity in young athletes. In this randomized, double-blind, and placebo-controlled study, ten highly trained male post-puberal futsal players aged 15.9 ± 1.2 years conducted two laboratory sessions. Athletes performed the Wingate test 60 min after ingestion of caffeine (CAF, 6 mg/kg body mass) or placebo (PL, dextrose) (blinded administration). Peak power, mean power, and the fatigue index were assessed. During the performance of the Wingate test, electromyographic (EMG) data were recorded from selected lower limbs muscles to determine the root mean square (RMS), mean power frequency (MPF), and median power frequency (MDPF) as frequency domain parameters and wavelet (WT) as time-frequency domain parameters. Caffeine ingestion increased peak (0.80 ± 0.29 W/Kg; *p* = 0.01; d = 0.42) and mean power (0.39 ± 0.02 W/Kg; *p* = 0.01; d = 0.26) but did not significantly affect the fatigue index (52.51 ± 9.48%, PL: 49.27 ± 10.39%; *p* = 0.34). EMG data showed that the MPF and MDPF parameters decreased and the WT increased, but caffeine did not have a significant effect on these changes (*p* > 0.05). Moreover, caffeine ingestion did not significantly affect RMS changes in the selected muscles (*p* > 0.05). Here we showed that acute caffeine ingestion improved anaerobic performance without affecting EMG parameters in young male futsal athletes.

## Introduction

Caffeine (1,3,7-trimethylxanthine) is a popular ergogenic aid that athletes use widely at all levels to improve sports performance [1, 2]. To date, much research has been done on the effectiveness of caffeine in sport and exercise performance [2–5]. Several studies have suggested that caffeine ingestion effectively improves aerobic endurance [3–5] and, to a lesser extent, short-term high-intensity anaerobic exercise performance [5, 6]. However, many factors can influence the ergogenic effects of caffeine use in sport and exercise performance, such as sex, time of day, genotype, habitual use, and training status [2]. Previous research shows some ambiguities regarding the physiological mechanisms underlying caffeine’s ergogenic effects [3, 5, 7]. Hardly anything is known on the neuromuscular effects of caffeine ingestion [7] following high-intensity, short-duration anaerobic exercise [5, 8–10].

Previous research suggests that caffeine affects the central nervous system (CNS) through the antagonism of adenosine receptors, leading to increased neurotransmitter release, motor unit firing rates, and pain suppression [4]. Davis et al. [11] suggested that caffeine can significantly delay fatigue through CNS mechanisms by blocking adenosine receptors. Among the cellular effects of caffeine, it has been observed that caffeine increases the availability of myofibrillar calcium, which facilitates force production by each motor unit [4]. Furthermore, fatigue, caused by the gradual reduction in calcium ion release, can be attenuated after caffeine ingestion [4]. Although the efficacy of caffeine on adenosine receptors and myofibrillar calcium ion mobilization is well-documented [4, 11], its effect on neuromuscular function and fatigue during short-term performance of anaerobic exercises cannot be observed, or recorded. Some studies have investigated the effect of caffeine ingestion on electromyographic (EMG) signal variables and neuromuscular fatigue during anaerobic exercise [8–10]. This research shows that caffeine does not affect EMG frequency parameters and fatigue. For example, Greer, Morales, and Coles [8] concluded that caffeine ingestion does not affect surface EMG frequency variables during a 30-second high-intensity Wingate test. Furthermore, Pereira et al. [9] reported that caffeine does not significantly change the fatigue index during the Wingate test. These latter investigators did not observe significant differences between males and females with regards to the rate of muscle fatigue. Juan et al. [10] additionally reported that caffeine supplementation significantly improved anaerobic performance without affecting EMG activity and fatigue levels. Therefore, there is some uncertainty about the physiological mechanism of caffeine ingestion on neuromuscular function during intense anaerobic power activities, such as the Wingate test performance. Furthermore, there is a literature gap on how caffeine consumption improves anaerobic performance [6, 10] without affecting fatigue indices [8–10].

Surface EMG frequency analysis is a standardized method to evaluate muscular fatigue during physical sport activities [12, 13]. Research shows that time-frequency analysis methods have higher accuracy rates versus time-dependent and frequency-dependent methods to detect muscular fatigue [14], especially during dynamic activities [15]. Among the studies mentioned above, only Pereira et al. [9] used the Fast Fourier Transform (FFT) parameter as a method for time-frequency analysis to investigate the effect of caffeine on neuromuscular fatigue during anaerobic exercise. Their results showed that caffeine consumption did not have a significant effect on muscle fatigue [9]. According to Vitro-Costa et al. [15], the wavelet transform (WT) is more efficient than the FFT during dynamic contractions [15]. They concluded that the WT is likely to be more adequate for dynamic tasks, as it requires no signal to be quasi-stationary, unlike the limitation imposed upon the use of the FFT [15].

In light of the prior discussion, this study aimed to investigate the effects of caffeine ingestion versus placebo on anaerobic performance and EMG frequency parameters during the Wingate anaerobic power test in young male futsal athletes. We hypothesized that the WT method might be more sensitive than the FFT method when examining the effects of caffeine on anaerobic performance and neuromuscular fatigue [15].

## Materials and methods

Ten youth male and highly trained futsal athletes aged 15.9 ± 1.2 years (body mass: 62.9 ± 8.8 kg, and body height: 169.8 ± 9.5 cm) volunteered to participate in this study and signed a written informed consent after information on benefits and risks of study participation were provided. Informed consent was obtained from all participants and their parent and/or legal guardian. The local ethical research committee approved this study (R.SSRI.REC.1400.1184).

A minimal sample size was calculated using an a priori statistical power tool (G∗Power, Version 3.1, University of Dusseldorf, Germany). The a priori power analysis was computed for our primary outcome (i.e., mean power [W]) with the following input parameters: assumed power of 0.80, alpha level of 0.05, medium effect size Cohen’s f = 0.52 [16]. The results of the power analysis indicate that 10 participants would be required to achieve significant group-by-time interactions.

To be eligible to participate in this study, individuals had to be (i) normal weighted as indicated by the body mass index (18–23 kg/m^2^), (ii) free of lower extremity injuries, and (iii) free of neuromuscular, cardiovascular, or mental illnesses [9, 10, 17]. Participants were diagnosed as post-pubertal by a physician using the Tanner and Whitehouse method and the stages of pubic hair development [18]. Habitual CAF consumption was determined by a questionnaire [19]. None of the participants consumed caffeine habitually. Daily caffeine consumption was less than 70 mg. Participants were excluded from the analysis if they did not have natural patterns of sleep (8-h/day on average), felt tired on the test day, and did not follow the dietary instructions 24 h before the test [17]. The standardized diet protocol did participants not allow to consume caffeinated beverages and foods such as coffee, cocoa, and dark chocolate [8]. In a randomized, double-blind, placebo-controlled study design, participants were scheduled to come in for two testing sessions, separated by 72 h [9]. Participants were instructed to ingest either caffeine (CAF, 6 mg/kg body mass) or placebo (PL, dextrose) supplements, which were concealed in opaque, unmarked containers. A trained laboratory expert provided the supplements to the athletes one hour before undergoing the Wingate test [8, 9].

The Wingate test was performed on a bicycle ergometer (Ergomedic 894E, Monark, Sweden). Participants were familiarized with the Wingate test procedures in a separate session prior to the actual test session. Before performing the Wingate test, participants warmed up for four minutes on a cycling ergometer at low intensity and 70–80 revolutions per minute (RPM), followed by dynamic stretching. After a three-minute rest period, the participant’s resistance on the ergometer was set at 7.5% of the body mass of the respective participant [9, 10]. Athletes were allowed to pedal for a few seconds until their pedaling speed reached 120 RPM, then the resistance was applied. Participants were verbally encouraged to pedal at maximum speed for 30 s. Peak power and mean power were the recorded anaerobic performance variables. The anaerobic performance variables and the fatigue index were calculated using Wingate software (Monark anaerobic test software, Sweden, 3.7.16) recorded during the 30 s Wingate test.

The Noraxon DTS wireless electromyography system (Noraxon DTS. USA) was used to detect myoelectric activity from the selected muscles during the Wingate test. To detect signals, a pair of Ag/AgCl electrodes (Model SKINTACT, Austria) with a diameter of 12 mm in dipole array was placed in the muscular belly of the selected muscles, including m. vastus medialis (VM), vastus lateralis (VL), rectus femoris (RF), gastrocnemius lateral head (GS) and biceps femoris (BF) of the dominant leg according to the European concerted action surface EMG for Non-Invasive Assessment of Muscles (SENIAM) [20]. The dominant leg was defined using the ball-kick test [21] the day before the first Wingate trial was performed. In the ball-kick test, players were kindly asked to kick a ball with moderate intensity and maximal accuracy through a set of cones placed 1 m apart and 10 m from the subject. The leg used to kick the ball was identified as the dominant leg [21]. Before surface electrode placement, the skin was shaved and cleaned with a pad soaked in 70% alcohol (Ethanol-C2H5OH) to reduce skin impedance. The signals were recorded at a sampling frequency of 1500 Hz, a gain of 500, CMR > 100 db, and a 10–500 Hz bandwidth filter to reduce electrical interference from external sources [22]. The EMG signal was processed with Noraxon software (version MR3 3.14.16). The EMG data detected during the 30-second Wingate test were divided into six 5-second epochs. The six epochs included data collected on the Wingate test at 0–5, 6–10, 11–15, 16–20, 21–25 and 26–30 s [9, 10, 23]. Normalized root mean square (RMS) was used to report the values of the EMG signal amplitude. The RMS data were normalized by the RMS values obtained during each muscle’s maximum voluntary isometric contraction (MVIC) [20]. Subjects performed two maximum voluntary isometric contractions for each muscle according to SENIAM recommendations (www.seniam.org) for five seconds with a one-minute break. An average of three seconds in the middle of the MVICs was used for normalization purposes [24].

The mean power frequency (MPF), the median power frequency (MDPF) and the WT were selected for frequency analysis. MPF and MDPF were calculated from the Eqs. 1 and 2.

1 $$ \rm{MPF}=\frac{{\int }_{{f}_{0}}^{{f}_{1}}f.PS\left(f\right)}{{\int }_{{f}_{0}}^{{f}_{1}}PS\left(f\right)}$$

where PS (f) is the EMG power spectrum and f_0_ and f_1_ stand for the surface electromyography bandwidth (f_0_ = lowest frequency and f_1_ = highest frequency of the bandwidth).

2 $$ \rm{MDPF}={\int }_{{f}_{0}}^{{f}_{median}}PS\left(f\right) df={\int }_{{f}_{median}}^{{f}_{1}}PS\left(f\right) df$$

where PS(f) is the EMG power spectrum and f_0_ and f_1_ stand for the surface electromyography bandwidth (f_0_ = lowest frequency and f_1_ = highest frequency of the bandwidth).

To calculate WT, we calculated one-dimensional wavelet decomposition with the type of Db4 mother wavelet of each window 5 s preprocessed signal with a sampling frequency = 1500 $\frac{samples}{s}$) and all the wavelet coefficients (approximate and detail coefficients) were extracted [15, 25, 26]. Thereafter, we reported the detailed coefficient of wavelet decomposition. The spectrum of the raw signal in each 5-second epoch of the 30-seconds Wingate test was normalized using related parameters, such as MPF, MDPF, and WT in the first epoch [9]. The calculations were performed using MATLAB software (2018a, The Mathworks Inc., Natick, MA, USA).

As noted, this study was conducted in a double-blinded, placebo-controlled, crossover design. Normal distribution of data was examined and confirmed using the Shapiro-Wilk test. The paired t-test was used to investigate the effects of caffeine ingestion on peak power, mean power, and the fatigue index. Furthermore, Cohen’s d effect sizes were used to highlight important pairwise differences, with values of 0.2, 0.5, and 0.8 corresponding to small, medium, and large effects, respectively [27]. The effect of caffeine on the parameters of the EMG signal was examined using a two-way (treatment [caffeine and placebo] × time [6-time epochs]) analysis of variance (ANOVA) with repeated measures. In case of significant treatment-by-time interaction effects, Bonferroni corrected post-hoc tests were computed. Statistical significance was accepted at *p* < 0.05. Statistical analysis was performed using SPSS software (version 25, IBM Corporation, Armonk, NY, USA).

## Results

Table 1 represents the values of the Wingate performance variables for both CAF and PL conditions. Caffeine but not placebo increased the peak power (t= -3.22; *p* = 0.01; d = 0.42) and the mean power (t= -2.295; *p* = 0.01; d = 0.26), and this increase was statistically significant. No statistically significant differences (t= -1.005; *p* = 0.34; d = 0.32) were observed with regards to the fatigue index under CAF and PL conditions.

**Table 1:** Mean values ± standard deviations for peak power, mean power, and the fatigue index in the CAF and PL conditions

	Peak power (W/Kg)	Mean power (W/Kg)	Fatigue index (%)
CAF	9.90 ± 2.04^*^	7.57 ± 1.38^*^	52.51 ± 9.48
PL	9.10 ± 1.75	7.18 ± 1.4	49.27 ± 10.39

CAF, caffeine; PL, placebo.

^*^ Indicates significant differences between conditions (*p* < 0.05).

**Fig. 1:**
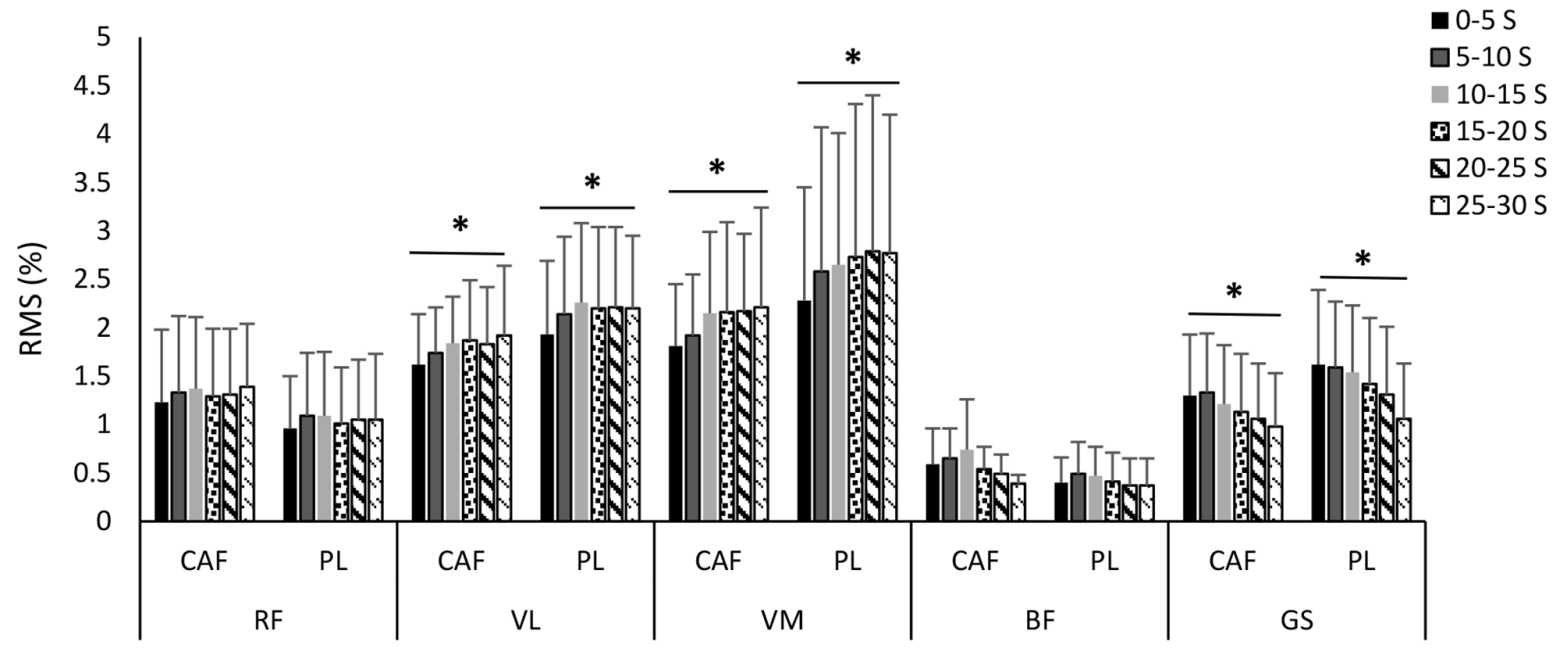
Mean and standard deviations for the normalized root mean square (%) of RF, VL, VM, BF and GS during six Wingate splits for all participants according to test condition (CAF and PL). RF, rectus femoris; VL, vastus lateralis; VM, vastus medialis; BF, biceps femoris; GS, Gastrocnemius; CAF, caffeine; PL, placebo; s, second. ^*^ Indicates significant differences over time (*p* < 0.05).

The mean and standard deviations of the normalized root mean square (%) for RF, VL, VM, BF, and GS during six Wingate test splits were analyzed for CAF and PL conditions and are illustrated below (Fig. 1). No significant differences were found for RMS of all muscles between CAF and PL. In both conditions, the RMS increased significantly over time in VL (F = 10.83; *p* = 0.01; d = 0.91) and VM (F = 7.07; *p* = 0.03; d = 0.88) muscles. RMS decreased significantly over time in GS (F = 7.69; *p* = 0.03; d = 0.91) under both conditions.

**Fig. 2:**
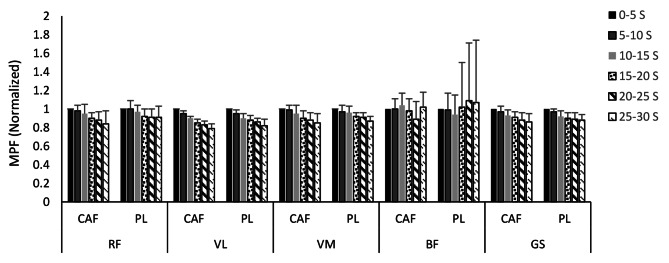
Mean and standard deviations of the normalized mean power frequency during six Wingate splits for all participants according to the test condition (CAF and PL). RF, rectus femoris; VL, vastus lateralis; VM, vastus medialis; BF, biceps femoris; GS, Gastrocnemius; CAF, caffeine; PL, placebo; s, second

The mean and standard deviations of the normalized mean power frequency of RF, VL, VM, BF, and GS during six Wingate splits in CAF and PL conditions are illustrated in Fig. 2. There were no significant differences in MPF between CAF and PL conditions in all muscles studied. In both conditions, MPF decreased significantly over time in all selected muscles (RF: F = 8.78; *p* = 0.001; d = 0.76, VL: F = 46.45; *p* = 0.001; d = 0.94, VM: F = 23.05; *p* = 0.001; d = 0.89, GS: F = 14.35; *p* = 0.001; d = 0.85), except for BF.

**Fig. 3:**
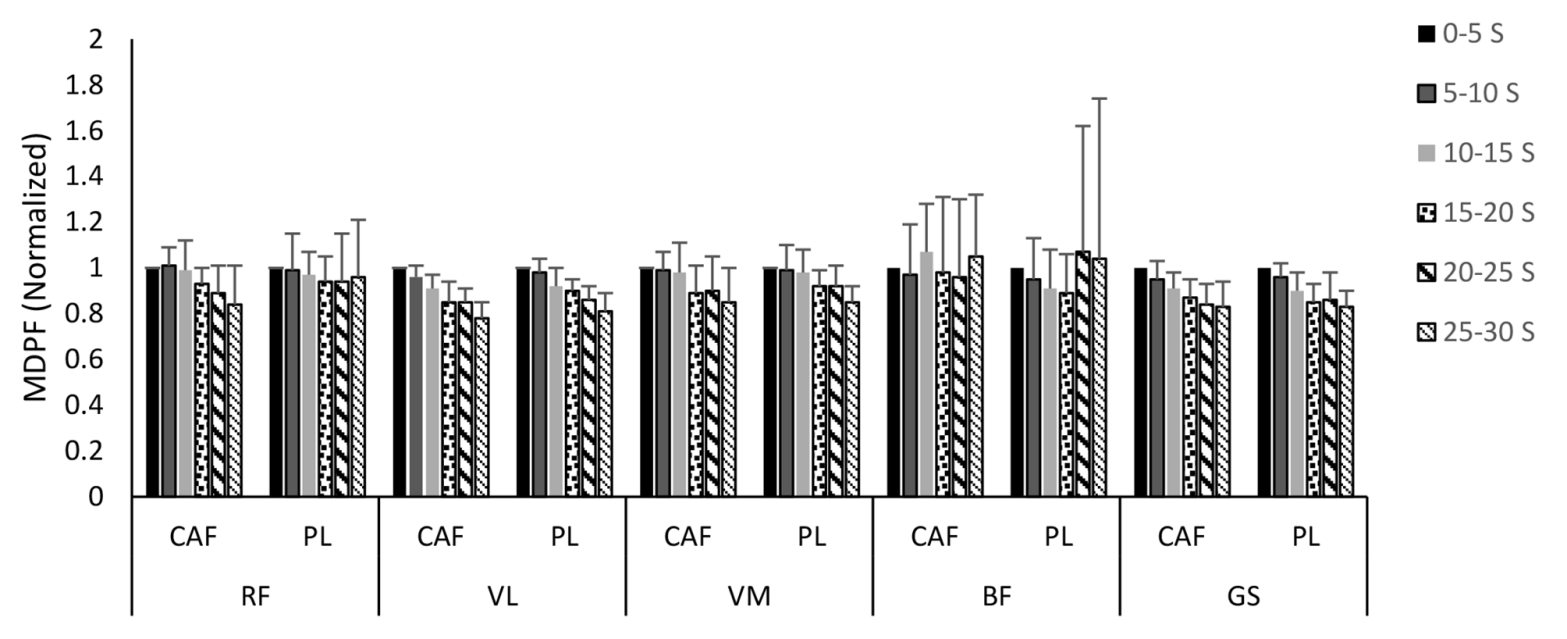
Mean and standard deviations of the normalized median power frequency of the RF, VL, VM, BF, and GS during six Wingate splits for all participants in the CAF and PL conditions. RF, rectus femoris; VL, vastus lateralis; VM, vastus medialis; BF, biceps femoris; GS, Gastrocnemius; CAF, caffeine; PL, placebo; s, second

The mean and standard deviations of the normalized median power frequency of RF, VL, VM, BF and GS during six Wingate splits according to CAF and PL conditions are illustrated in Fig. 3. There were no significant differences in MDPF between CAF and PL conditions in all muscles studied. In both conditions, MDPF decreased significantly over time in the VL, VM and GS muscles (VL: F = 32.64; *p* = 0.001; d = 0.92, VM: F = 14.13; *p* = 0.001; d = 0.83, GS: F = 13.33; *p* = 0.001; d = 0.84), but these changes were not significant in the BF and RF muscles.

**Fig. 4:**
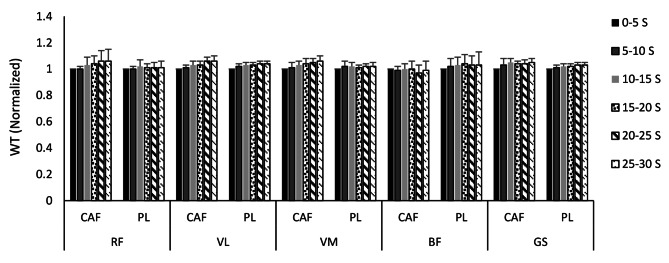
Mean and standard deviations of the normalized Wavelet transform for RF, VL, VM, BF, and GS during six Wingate splits according to CAF and PL conditions. RF, rectus femoris; VL, vastus lateralis; VM, vastus medialis; BF, biceps femoris; GS, Gastrocnemius; CAF, caffeine; PL, placebo; s, second

The mean and standard deviations of the normalized Wavelet transform during six Wingate splits of five selected muscles under the CAF and PL conditions are illustrated in Fig. 4. In both conditions, WT increased significantly (*p* < 0.05) over time in all selected muscles (VL: F = 11.11; *p* = 0.001; d = 0.80, VM: F = 6.97; *p* = 0.001; d = 0.71, GS: F = 11.39; *p* = 0.001; d = 0.80), except for RF and BF. WT frequency measures did not differ significantly (*p* > 0.05) for any muscles evaluated between the CAF and PL conditions.

## Discussion

The results of this study indicate that caffeine consumption increases peak power and mean power in highly trained post-pubertal futsal players during the Wingate test but has no effect on the fatigue index. EMG signal analysis showed that the MDF and MDPF frequency domain parameters decreased, while the WT time-frequency domain parameter increased. However, caffeine did not have a significant effect on these changes. The value of RMS in the RF and BF muscles did not change during the Wingate test. However, RMS increased in the VL and VM muscles while GS muscle activity decreased during the 30-second Wingate test. Caffeine ingestion did not have a significant effect on changes in RMS of the selected muscles.

Regarding the effects of caffeine ingestion on anaerobic performance measures, our results are consistent with those of San Juan et al. (2019), who examined professional athletes [10] but contrary to Greer et al. [8] and Pereira et al. [9]. They investigated recreationally active individuals [8, 9]. Surveys show that the ergogenic effects of caffeine on exercise performance appear to be affected by the training status of individuals [2]. Although the physiological reasons for this are unclear, Mizuno et al. [28] reported that the densities of adenosine A_2A_ receptor numbers are higher in trained individuals than in untrained individuals [28]. Caffeine competes with adenosine receptors in the CNS, heart, and skeletal muscle [4, 28]. These receptors include A_2A_, A_2B_, A_3,_ and A_1_. Studies show that caffeine is more effective in A_1_ and A_2A_ [4]. Adenosine A_2A_ receptors in skeletal muscle help regulate glucose uptake and contractile force [28]. Due to the higher density of A_2A_ in the muscles of trained individuals, the binding of caffeine to these receptors and subsequent improvements in exercise performance is likely increased in trained individuals [28]. Although the results of research on the effectiveness of caffeine consumption on anaerobic performance are somewhat contradictory [8–10], a meta-analysis by Grgic et al. [6] showed that caffeine consumption improves peak power and mean power during Wingate testing [6].

The lack of effect of caffeine consumption on neuromuscular fatigue and frequency of the EMG signal in this present study was consistent with the results of two previous studies [8, 9]. In the present study, we concluded that caffeine does not affect the amplitude of the EMG signal, which is consistent with previous literature [8, 10]. According to previous studies, the impact of caffeine on CNS activity can increase neural drive to muscles, increase neurotransmitter release in the CNS, increase motoneuronal excitability, and increase motor unit recruitment [29]. However, in agreement with previous studies [8, 9], the present study does not support the hypothesis of increased motor unit recruitment and firing rate during supramaximal anaerobic exercise. Although caffeine may improve peak power and mean power during the Wingate test, it does not appear to affect motor unit recruitment and firing rate in this present study. Therefore, the ergogenic effect of caffeine on anaerobic performance appears to be peripheral, which is consistent with Greer et al. [8].

An important finding in the present study, confirmed by previous research [28], is that caffeine ingestion had no effect on signal amplitude in the selected muscles evaluated in this study. The RMS amplitude decreased in the GS muscle, which was in line with the results found in San Juan et al. [10], and increased in the VL and VM muscles. The variation of signal amplitude changes in different muscles during the Wingate test needs to be investigated.

The mechanism of the effect of caffeine on CNS function during short-term high intensity exercise is not yet well understood [5, 7]. However, this lack of effect may be related to the nature of the Wingate test. During the 30-second all-out exercise test, high-frequency fast-twitch motor units are recruited. With fatigue, the firing rate decreases to ensure protection of whole-body homeostasis [30]. Caffeine consumption causes an increase in neural drive to muscles, increased release of neurotransmitters into the CNS, and eventually increased CNS activity [29]. However, it should be noted that the CNS is unique as it attempts to save a person from injury. The CNS may attenuate the firing frequency of motor units to prevent possible muscle injury (the concept of muscle wisdom) [31]. If the duration of the test were longer, perhaps the effect of caffeine on CNS function would be apparent and the CNS would delay fatigue by recruitment of subsequent motor units [23]. Hunter et al. (2003) found that healthy men who perform the Wingate test in a 30-second period are ineffective for the feedback loop between intramuscular metabolism and the CNS to affect motor unit recruitment strategies [23].

Yousif et al. [32] stated that fatigue detection would be more accurate if the amplitude of the EMG signal were combined with other methods, such as spectral analysis, to obtain better detection and more reliability [32]. Since the WT analysis showed that caffeine has no effect on fatigue and neuromuscular function, we can interpret that the ergogenic mechanisms of caffeine appear to be peripheral and not central due to the WT taking into account the frequency and amplitude of the signal together to identify potential fatigue. Therefore, the ergogenic effects of caffeine to improve anaerobic power during the 30-second time frame of the Wingate test are not related to its effect on the CNS but to peripheral mechanisms [8].

This study is not without limitations. Our study involved only male participants, raising questions about the applicability of the results to female populations, making it difficult to extrapolate the findings to females. To address these limitations and enhance the robustness of future research, there is a clear need for larger-scale studies that includes both mixed-gender and female populations.

## Conclusions

The results of our study provide evidence that acute caffeine ingestion improved anaerobic performance (Wingate test) in young male futsal players. Given that participants of the present study were highly trained young futsal players and the nature of this sport requires aerobic and anaerobic energy pathways throughout exercise, cellular adaptations in the muscle tissue of these athletes may provide the conditions for the absorption of peripheral ergogenic effects of caffeine. Future studies are recommended to compare the effects of caffeine consumption on nerve firing rate and CNS function during intense short-term activity between professional and amateur athletes.

## Data Availability

The datasets generated during and analyzed during the current study are not publicly available due to confidential information about the participants but are available from the corresponding author on reasonable request.
